# Metamaterials for simultaneous acoustic and elastic bandgaps

**DOI:** 10.1038/s41598-021-94053-3

**Published:** 2021-07-19

**Authors:** Waiel Elmadih, Dimitrios Chronopoulos, Jian Zhu

**Affiliations:** 1 Institute for Aerospace Technology & The Composites Group, University of Nottingham, Nottingham, NG8 1BB UK; 2 Metamaterials Ltd, Wallington, SM6 0TL Surrey UK; 3 Department of Mechanical Engineering & Mecha(Tro)Nic System Dynamics (LMSD), KU Leuven, Ghent Technology Campus, 9000 Leuven, Belgium; 4 School of Mechanical Engineering & State Key Laboratory for Strength and Vibration of Mechanical Structures, Xi’an Jiaotong University, Xi’an 710049, China

**Keywords:** Condensed-matter physics, Soft materials, Structural materials, Techniques and instrumentation, Mechanical engineering

## Abstract

In this work, we present a single low-profile metamaterial that provides bandgaps of acoustic and elastic waves at the same time. This was done by ensuring impedance mismatch in two different domains, the fluid domain where the acoustic waves propagate and the solid domain where the elastic waves propagate. Through creatively designing the metamaterial, waves of certain nature and frequencies of interest were completely blocked in the solid and fluid domains simultaneously. The simulation results showed bandgaps with acoustic waves attenuation below 5 kHz and elastic waves attenuation below 10 kHz. The acoustic and elastic dispersion curves of the metamaterials were calculated for various designs with various diameters and neck lengths, and the bandgaps were calculated. These parameters can be used as means for tuning both the acoustic and elastic bandgaps. A representative design of the metamaterial was manufactured on a laser powder bed fusion system and the dynamic performance was measured at various points. The measurements were carried out using a dynamic shaker setup and the dynamic performance was in good agreement with the numerical modelling results. Such metamaterials can be used for simultaneous acoustic and elastic attenuation, as well as saving in space and material consumption, in various fields including building construction, automobile, aerospace and rocket design.

## Introduction

With their broad range of properties, metamaterials have been serving as a lightweight solution for many dynamic, static and electromagnetic applications. Through harnessing certain topologies of the micro- and macro-structures of the material, metamaterials can create bandgaps of either acoustic waves, elastic waves or electromagnetic waves independently. There exist a chance for creating metamaterials that exhibit various bandgaps of different waves at the same time. The acoustic and elastic bandgaps are concerned with different types of waves as their names suggest. The acoustic bandgaps are formed by acoustic waves; these waves are oscillations of pressure travelling through a fluid. The elastic bandgaps are formed by elastic waves; these waves are formed by disturbances travelling through a solid. Both elastic and acoustic bandgaps are formed by the destructive interference of their waves^1–3^. When a wave travels from one medium to another of less local impedance, for example, an acoustic wave changing its speed when moving from a thin neck to a larger cavity, some of the waves get reflected. When a reflected wave is in phase with the travelling wave, both waves interfere destructively with each other. This destructive interference of the reflected wave and the travelling wave results in the creation of bandgaps.

The realisation of structures that have bandgaps has been made available through additive manufacturing (AM) which is a manufacturing method that can construct complex parts from a CAD model. Many AM techniques exist of which the most common are extrusion-based AM^4^, laser powder bed fusion (LPBF)^5^ and stereolithography^6^. These techniques have been used to develop various bandgap structures by incorporating Bragg-scattering and internal resonance capabilities within the geometry of the designed structures. Warmuth et al.^7^ designed and manufactured lattices made from connected struts and demonstrated elastic bandgaps above 50 kHz. Lucklum et al.^8^ used strut-based lattices to create structures with three-dimensional (3D) elastic bandgaps in the millimeter scale. Kruisova et al.^9^ built ceramic lattices and experimentally demonstrated the existence of one-dimensional (1D) elastic bandgaps. Multimaterial lattices have also been investigated by Ampatizids et al.^10^ who built a composite lattice made of a Nylon-12 part attached to a flat composite sheet; they demonstrated with experiment the existence of 1D bandgaps below 10 kHz. Liu et al.^11^ developed lattices, made of solid cores and a coating of silicone rubber, and managed to obtain elastic bandgaps at much lower frequencies than that of Warmuth et al.^7^, Lucklum et al.^8^ and Ampatizids et al.^10^ through building an internal resonance mechanism within the lattice. Recent work by Lucklum et al.^12^ also employed an internal resonance mechanism and showed that lattices could have 3D elastic bandgaps at low-frequencies. Cubic lattice structures with internal resonances were developed and tested by Elmadih et al.^13^ who conclude that their design can obtain 3D elastic bandgaps of wide- and low-frequency range. On the acoustic side, Abueidda et al.^14^ demonstrated with a dynamic simulation that triply periodic minimal surface (TPMS) lattices can have acoustic bandgaps. TPMS lattices were also featured in the elastic bandgap work of Elmadih et al.^15^ and the mechanical work of Maskery et al.^16^. Bilal et al.^17^ presented an architecture metamaterial with vibration attenuation capabilities of airborne sound and vibration simultaneously. Hsu et al.^18^ designed and simulated a crystal strip waveguide with acoustic bandgaps. Lazcano et al.^19^ localised the acoustic modes in a lattice structure to develop acoustic bandgaps. Jiang et al.^20^ designed and simulated an architected 3D foam with acoustic and elastic bandgaps.

Metamaterials with acoustic and elastic bandgaps are essential for producing compact, lightweight and efficient sound and vibration isolation solutions for use in space rockets, cars, machines and buildings. This will lead to massive savings in time and effort associated with machine assembly and building construction. This means that the capabilities to provide vibration and acoustic isolation can now be superior to the conventional ones by using the presented metamaterial design. This design can be embedded within the structure of the part rather than having to use two separate bulky mechanisms; one for vibration isolation and one for acoustic isolation. For example, to reduce the noise and vibration radiated from a wall, a designer would have to develop and use a membrane-plate/porous metamaterial for sound isolation and a phononic crystal for mechanical vibration isolation^21–23^, seperately; this design would be complicated, expensive and bulky. The intrinsically lightweight nature of metamaterials will provide extra savings in mass in comparison to conventional designs, thus allowing for higher mobility and flexibility in manufacturing and building construction. For instance, these metamaterials can be used for reducing acoustic and mechanical vibration waves arising from electric motors that are used to power airplanes. They can also be used for isolating the workpiece and end effector from noise and perturbations during manufacturing and measurement processes.

**Figure 1:**
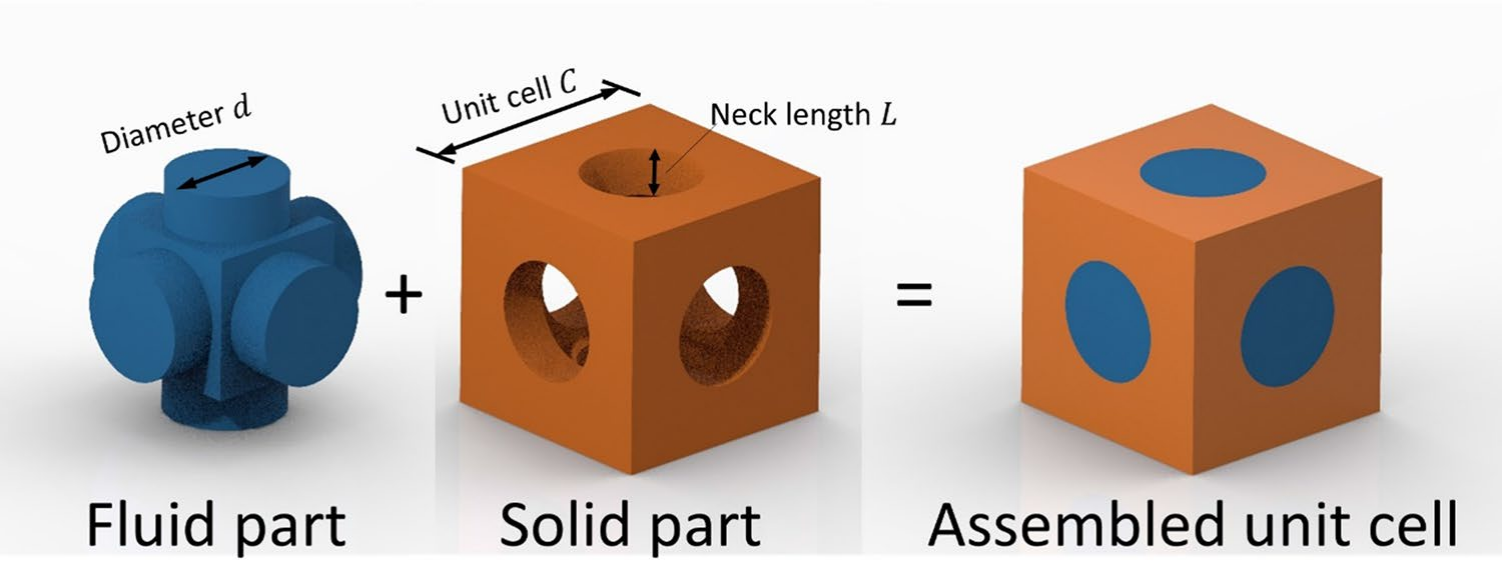
Design of the metamaterial unit cell.

In this work, we present a cubic metamaterial design inspired by the Primitive form of the TPMS lattice, featured in various mechanical and vibration work^15,24–26^, with verified acoustic and elastic bandgaps. The microstructure of the metamaterial consists of a unit cell, shown in Fig. 1, with a fluid domain where the acoustic waves propagate and a solid domain where the elastic waves propagate. The fluid domain is shaped as a cubic resonator of neck length $L$ and and neck diameter $d$. The solid domain of macrostructure of the metamaterials is a single cubic lattice with a unit cell size $C$. The manufacturing of such a unit cell can be made easily through various forms of AM techniques, for example, L-PBF^27^, two-photon lithography^28^, and fusion deposition modelling (FDM)^4^. Details of the computation, manufacturing and experimental testing methods are provided in the Methods section towards the end.

## Results and discussions

The metamaterial unit cell shown in Fig. 1 was designed using Computer-Aided Design (CAD). For the acoustic absorption to be effective, an interconnected pore is needed within the design to allow for the passing of air. That is why the design of the metamaterial is an open-pore structure^29^. The dimensions of the geometrical features of the unit cell are expressed as ratios in reference to the unit cell size in Table 1.

**Table 1:** Dimensions of the main geometrical features of the unit cell normalised to the unit cell size $C$*.*

Design	$d/C$	$L/C$
Design 1	0.25	0.1
Design 2	0.5	0.1
Design 3	0.75	0.1
Design 4	0.25	0.2
Design 5	0.5	0.2
Design 6	0.75	0.2
Design 7	0.25	0.3
Design 8	0.5	0.3
Design 9	0.75	0.3

Each unit cell was meshed using a finite element modelling (FE) package using the proper couplings between the fluid and solid parts. The FE approach was used because it can simulate complex geometrical features more accurately with higher computation efficiency than finite difference time domain (FDTD)^30^ method, plane wave expansion (PWE)^31^ method and wavelet method^32,33^. Bloch’s theorem and periodic boundary conditions were used to compute both acoustic and elastic dispersion curves of the metamaterial. At first, the acoustic dispersion curves of the metamaterial were modelled using the governing equation of acoustic wave propagation in the frequency domain (see Methods section). Then, the elastic dispersion curves were computed by solving the eigenvalue equations constructed from the general harmonic equation of the infinite metamaterial. The acoustic and elastic response of the finite metamaterial was simulated using the FE package. A metamaterial with a finite length was additively manufactured using LPBF and the experimental elastic response was obtained.

The modelling of the bandgaps used the FE method while ensuring convergence of the results with respect to the mesh density per unit cell. The fluid part and the solid part were designed and assembled in CAD. The linear $x-$,$y-$, $z-$ and pressure degrees of freedom (DoF) of the converged mesh lattice were extracted using ANSYS modelling software. The converged mesh lattice is the lattice model that has sufficient number of mesh elements that produce accurate results. The linear DoF are associated with the solid part while the pressure DoF are for the fluid part.

**Figure 2:**
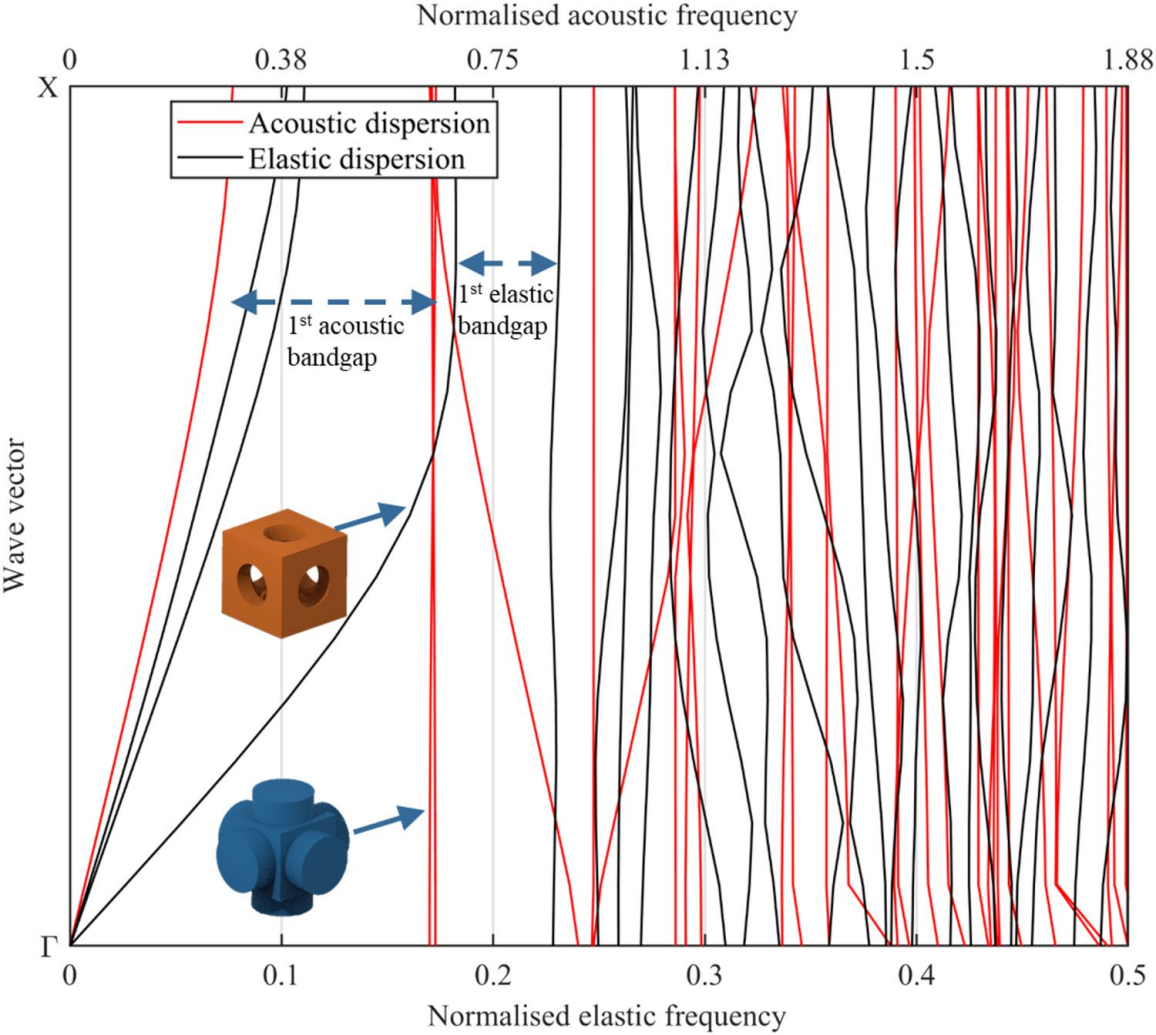
Acoustic and elastic dispersion curves of the metamaterial with annotations of the first bandgaps. The parameters used for the modelling are $d/C=$ 0.5, $L/C=$ 0.2 and a Poisson’s ratio $=$ 0.33.

The dispersion curves of the acoustic and elastic wavebands are presented in Fig. 2 for a metamaterial with a diameter to cell size ratio ($d/C$) of 0.5 and a neck length to unit cell size ratio ($L/C$) of 0.2. The acoustic frequency results are normalised to the unit cell size $C$ and speed of sound in the fluid ${v}_{f}$, while the elastic frequency results are normalised to the unit cell size $C$ and the longitudinal wave speed in solid ${v}_{s}$. The $y-$axis in Fig. 2 represents the wave vector; with Γ being the center of the irreducible Brillouin zone of the simple cubic lattice with coordinates (0,0) and X being the center of the face in a simple cubic lattice with coordinates ($\pi /C$,0). The wave vector could then be used to get the wavenumber at the critical points Γ and X of a metamaterial by substituting the value of the length of the unit cell $C$ in meters to get the wave number in units of m^−1^. From the results provided in Fig. 2, it can be seen that the metamaterial has multiple acoustic and elastic bandgaps at various frequencies.

**Figure 3:**
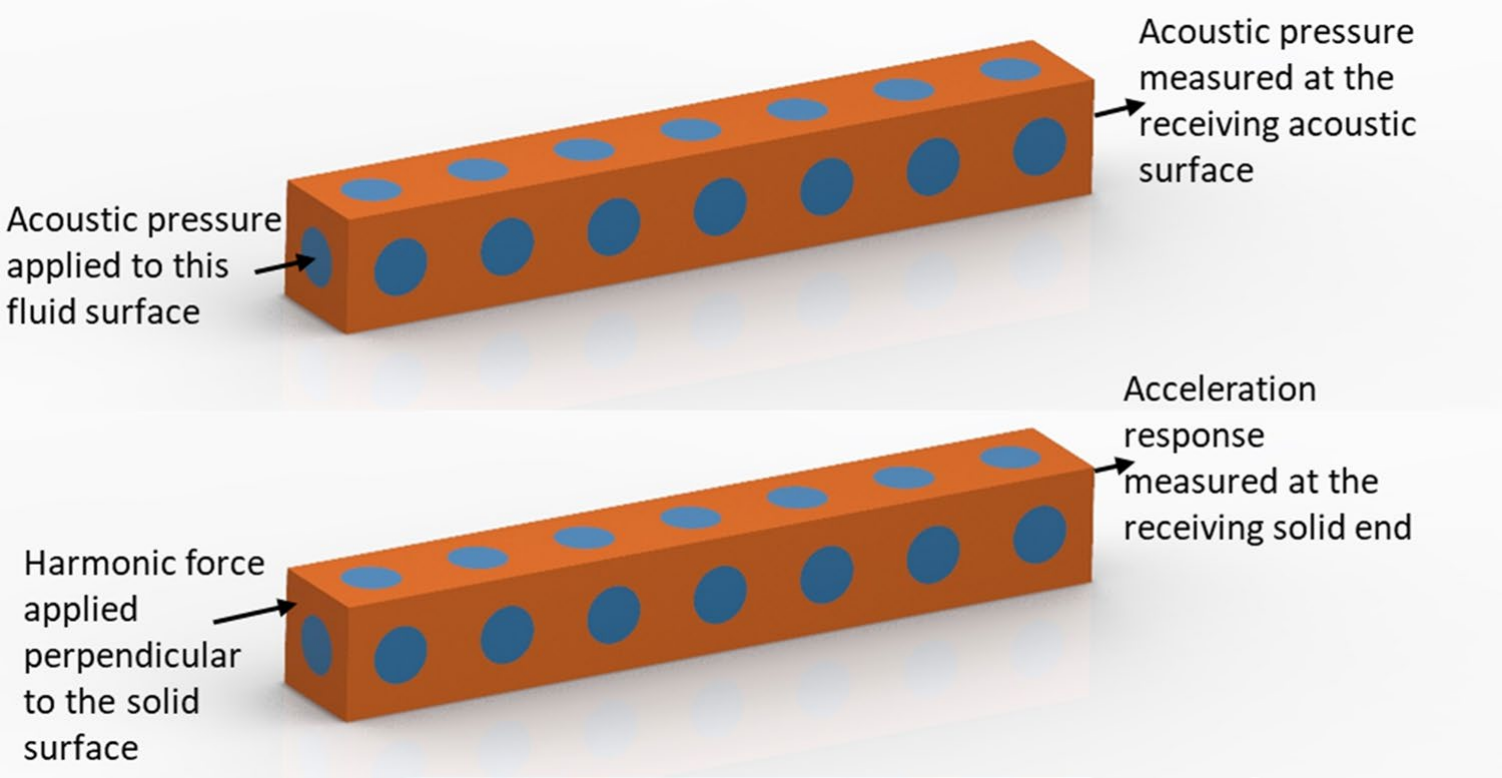
Illustration of the receiving and sending ends in acoustic simulation (top) and elastic simulation (bottom) of a finite-length metamaterial.

**Figure 4:**
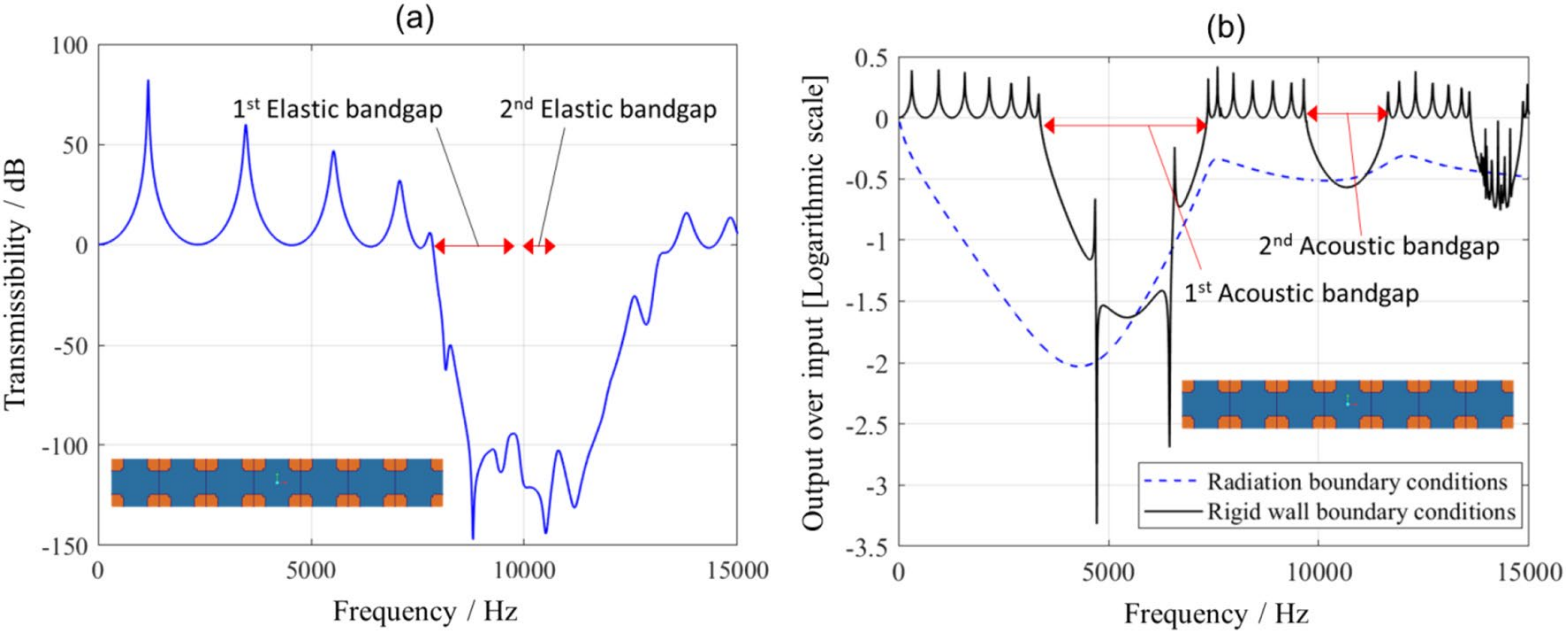
Simulation results of the ability of the metamaterial to transmit (**a**) elastic waves and (**b**) acoustic waves.

A useful metric for evaluating elastic and acoustic bandgaps is the relative bandgap width. The relative bandgap width gives an idea about the width and the position of the bandgap in terms of frequency. The relative bandgap is expressed as a percentage and is calculated as the quotient of the width of the bandgap to the mid-frequency of the bandgap. The first acoustic bandgap, appearing in Fig. 2, starts from a normalised frequency of 0.2911 to 0.6354 and has a relative bandgap width of 74%, while the first elastic bandgap spans a relative bandgap width of 27.4% from 0.1926 to 0.2538. Within the tested normalised frequency range from 0 to 1.88 in the acoustic frequency case and from 0 to 0.5 in the elastic frequency case, a total of five acoustic bandgaps and three elastic bandgaps exist in the presented metamaterial, respectively. These bandgaps can be tuned to a frequency of interest through the selection of the appropriate solid and fluid materials, as well as the unit cell size. For example, for a metamaterial manufactured from Nylon12 on a laser powder bed fusion (LPBF) system with a unit cell size of 30 mm, the longitudinal wave speed in solid is 1322.8 m s^−1^ and the speed of sound in air is 346.25 m s^−1^. Using the results from Fig. 2, the first acoustic bandgap for this metamaterial is from 3.36 kHz to 7.33 kHz and the first elastic bandgap is from 7.92 kHz to 9.84 kHz. To computationally verify the existence of these bandgaps, a simulation has been set up to depict the elastic and acoustic attenuation achievable with the presented metamaterial. The metamaterial shown in Fig. 3 has a unit cell size of 30 mm and was modelled with the properties of Nylon 12 for the solid domain and the properties of air for the fluid domain. A total of seven unit cells were used in the simulation, thus constructing a 7 × 1 × 1 metamaterial with a total finite length of 210 mm as shown in Fig. 3. The transmission of the acoustic and elastic waves has been attenuated in frequency regions that correspond well with that of the bandgaps of the metamaterial as can be seen in Fig. 4. On the elastic simulation side, a longitudinal harmonic force of 1 N was applied perpendicular to the solid surface of the metamaterial.

The vibration modes of the nearly flat wave branches (see Fig. 2) that split the first two elastic bandgaps do not get activated with longitudinal excitation. Thus, the two elastic bandgaps are merged into one bandgap in the simulation of the longitudinal elastic response of the metamaterial shown in Fig. 4a. On the acoustic side, radiation boundary conditions were used on the fluid openings in the direction of no tessellations (see Methods section). At first, an incident wave was sent through the fluid opening along the direction of tessellation of the metamaterial as shown in Fig. 3. We also show with the acoustic simulation in Fig. 4b that applying rigid wall boundary conditions on the fluid openings in the opposite direction to the direction of tessellation does not interfere with the existence of the acoustic bandgap. When radiation boundary conditions are used, there is no reflection at the fluid openings of the metamaterials. This allows the acoustic waves to travel to the outer environment of the metamaterial and, thus, less overall acoustic pressure will be recorded as can be seen in Fig. 4b. This means that the metamaterial can be used to attenuate elastic and acoustic vibrations in any application that has an open or closed fluid setup.

**Figure 5:**
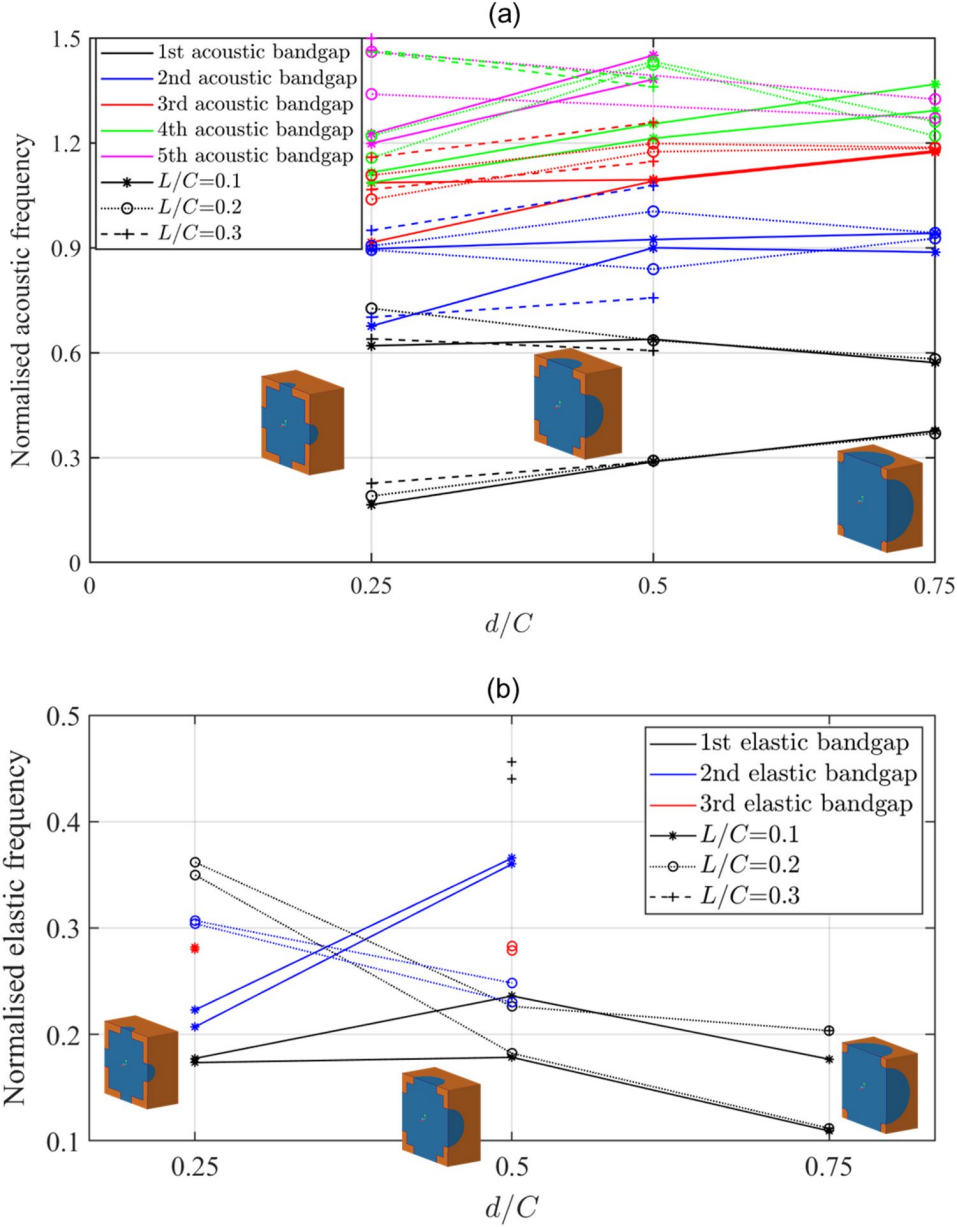
The change in the (**a**) acoustic and (**b**) elastic bandgaps of the metamaterial at various $d/C$ and $L/C$ ratios with illustrations of the cross-sectional view of the single unit cell. The bandgap is represented with a pair of identical lines, with the bottom line denoting the start frequency of the bandgap and the top line denoting the end frequency of the bandgap.

In addition to the unit cell size, solid material and fluid material, the geometrical features of the metamaterial can be used to control the width and position of the bandgap frequency range. Figure 5 shows the change in the acoustic and elastic bandgaps of the metamaterials when different $d/C$ and $L/C$ ratios are used in the construction of the metamaterial. These bandgap results were calculated from the dispersion curves of the metamaterials modelled with the parameters shown in Fig. 5.

The various geometrical parameters of the metamaterial can be used for tailoring the acoustic and elastic bandgaps for use in applications of known frequencies of interest. In general, a metamaterial with a higher $d/C$ ratio has a wider elastic bandgap of lower starting frequency than its counterparts of lower $d/C$ ratio. The lower starting frequency is mainly due to the lower dynamic stiffness of metamaterials of higher diameter opening. This is analogue to findings found in other publications^34–36^ where internal resonators are used to hinder wave propagation. In internal resonance, the lower the stiffness of the internal resonance mechanism, the lower is the starting frequency of the bandgap. However, not all internal resonance bandgap metamaterials are suitable for broad isolation frequency, unlike the broad bandgaps presented in this work, and only a hand full of publications^12,13,37^ showed capabilities to provide broad elastic bandgaps at lower frequencies but with no reference to its acoustic capabilities. The acoustic bandgap frequency ranges of the metamaterial presented in this work have a wider bandgap when a lower $d/C$ ratio is used as can be seen in Fig. 5a.

**Figure 6:**
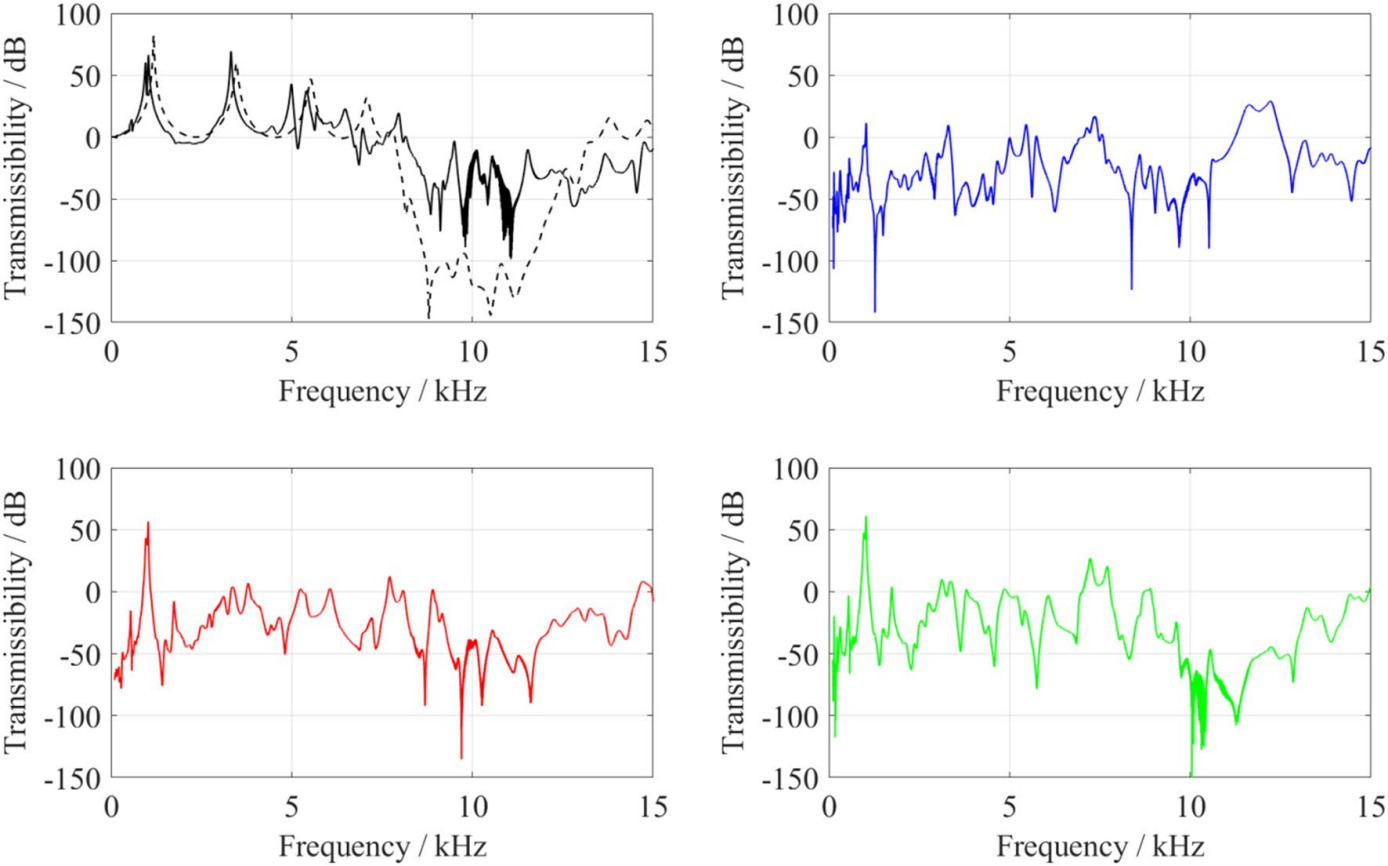
Experimental response of the manufactured metamaterials in (**a**) the longitudinal direction (solid line), in comparison to simulation with 2% damping factor (dashed line), and the cross-receptence response after the first (**b**), fourth (**c**) and seventh (**d**) periods of the metamaterial.

The elastic experimental response of the metamaterial was measured using a dynamic shaker and a set of piezoelectric accelerometers. The transmissibility was calculated as the logarithmic scale of the output response to the input response multiplied by a factor of 20 and is presented in Fig. 6 (see Methods section). It can be seen that the response of the structure is close to or much higher than 0 dB away from the frequency ranges of the bandgaps. At the bandgap, the experimental response gets as low as − 90 dB. This behaviour is similar to that shown by the simulation results with a damping factor of 0.02. However, in the simulation, the two bandgaps are shown as if they were merged into one wide bandgap. This is due to the nature of the simulation in which the mode shape between the end of the first bandgap and the start of the second bandgap was not sufficiently excited. In the cross-receptance results, the average attenuation along the bandgap saw a much lower response than the longitudinal one. The attenuation within the bandgap was proportional to the spatial periodicity (number of unit cells) between the excitation point and measurement point. Within the bandgap, the cross-receptence response has an average of − 31 dB at the first unit cell. This average response is even lower at the fourth unit cell, due to further destructive interference of the travelling waves occurring between the first and the fourth unit cells, and is equal to − 38 dB. At the end of the structure, at the seventh unit cell, further destructive interference occurs due to the periodicity and the average response gets as low as − 46 dB.

These results provide a new set of metamaterial structures that are capable of producing acoustic and elastic attenuation frequency regions. These attenuation regions are made available through the bandgap mechanisms designed within the presented metamaterial to design quieter, more stable and more damage resistant mechanisms. Stability is particularly important in precision engineering and machine design where the workpiece is required to stay stable during manufacturing so that parts are made more precisely and more accurately. The damage resistance capability is particularly important in building construction where excitations from nearby traffic and rotary components can lead to creep propagation and failure of the construction in the long-term. Simultaneously, the use of the acoustic-blocking capabilities of the metamaterial will also lead to quieter living spaces for humans and animals.

The attenuation of vibration and noise is essential for the performance of machines and buildings as well as the comfort of the occupants and workers. In this work:

- We presented a new metamaterial that can develop bandgaps of both acoustic waves and elastic waves. This was done using a finite element method employed for modelling the acoustic and elastic dispersion curves of the metamaterial.
- The acoustic and elastic bandgaps can be tailored to meet certain frequency ranges of interest through the selection of the appropriate material, cell length, neck length and diameter.
- The most significant bandgaps are the first acoustic bandgap and first elastic bandgap, due to their broader frequency ranges and higher mid-frequency in reference to the other bandgaps demonstrated by the same metamaterial.
- A material with a higher diameter to cell size ratio results in a wider first elastic bandgap and a narrower first acoustic bandgap, and vice versa. This can be used as a tool that gives more design freedom when tailoring the metamaterial for isolation of noise and vibration in an application of interest.
- Experimental results further verified the existence of the elastic bandgap in the presented metamaterial.
- These results can be used in manufacturing and building constructions as compact and lightweight solutions for high attenuation of acoustic and vibration waves.
- Future work will include testing the acoustic response of the metamaterial using an acoustic impedance tube of suitable size and characteristics.

## Methods

### Acoustic dispersion curves

**Figure 7:**
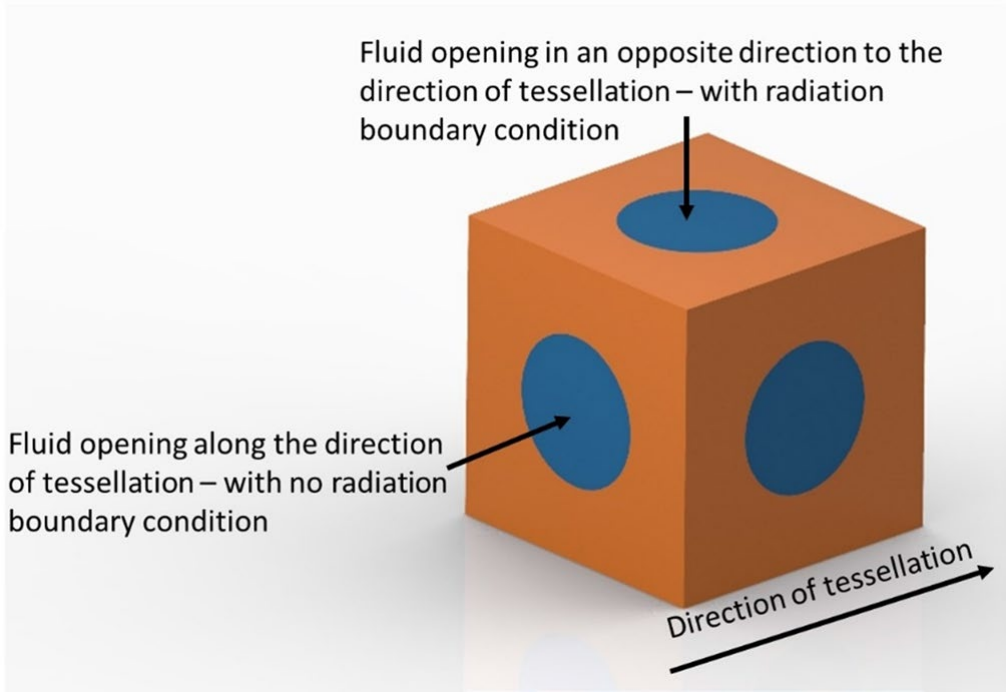
Radiation boundary conditions applied to the unit cell.

Acoustic wave propagation occurs mainly in the air (the fluid domain of the lattice). In reference to acoustic energy lost through harmonics of the solid material, the losses caused by the fluid that moves within the interconnected pores of the metamaterial is much higher. This energy loss is due to viscous boundary layer effects; since air is a viscous fluid, energy is lost through friction with the solid walls of the pores^29^. In its reduced form, the governing equation of acoustic wave propagation is obtained using^14^

1 $$\nabla \cdot \left(\frac{-1}{\rho }\nabla p\right)+\frac{{\omega }_{a}^{2}p}{{v}_{a}^{2}{\rho }_{a}}=0,$$

where ${\rho }_{a}$ is the density of the acoustic media, $p$ is the acoustic pressure, ${\omega }_{a}$ is the angular frequency of the acoustic wave and ${v}_{a}$ is the speed of sound in the fluid medium. The tessellation of the unit cell was assumed along one direction using Floquet periodic boundary conditions^38^, while wave propagation is allowed in three-dimensions (3D) using 3D mesh nodes for the metamaterial. Radiation boundary conditions were used on the fluid openings that are in opposite direction to the tessellation direction as in Fig. 7. This provides an accurate dynamic simulation of an infinite boundary of fluid away from these surfaces, thus ensuring acoustic waves are not reflected at these surfaces and negatively affecting the acoustic transmission results.

The periodic boundary conditions can be approximated using

2 $$p\left(x+C\right)=p\left(x\right){e}^{i{k}_{x}C},$$

where $p\left(x\right)$ is the pressure at position $x$, $p\left(x+C\right)$ is the pressure at the position obtained by a shift equal to the size of the unit cell $C$, $i$ denotes the imaginary unit and ${k}_{x}$ is the wavenumber along the direction of tessellation. By substituting Eq. (2) into Eq. (1) and extracting the stiffness and mass matrices of the unit cell from the FE model, we arrive at the eigenvalue problem

3 $${{\varvec{K}}}_{{\varvec{a}}}-{{\varvec{\omega}}}_{{\varvec{a}}}^{2}{{\varvec{M}}}_{{\varvec{a}}}=0,$$

where ${{\varvec{K}}}_{{\varvec{a}}}$ is the acoustic stiffness matrix and ${{\varvec{M}}}_{{\varvec{a}}}$ is the acoustic mass matrix. ${\varvec{K}}{}_{{\varvec{a}}}$ and ${{\varvec{M}}}_{{\varvec{a}}}$ are constructed for predetermined values of the wavenumber to sweep the edges of the irreducible Brillouin zone of the metamaterial from ${k}_{x}=$ 0 to ${k}_{x}=\pi /C$. By solving the eigenvalue problem in Eq. (3), constructed by each pair of ${\varvec{K}}{}_{{\varvec{a}}}$ and ${{\varvec{M}}}_{{\varvec{a}}}$, we get the frequencies of the waves propagating in the metamaterial which can be plotted in the form of acoustic dispersion curves. The absolute acoustic frequency ${f}_{a}$, plotted on the $x-$ axis of the dispersion curves, is normalised to the unit cell size $C$ and the speed of sound in the fluid medium ${v}_{f}$ using.

4 $$\mathrm{Normalised\:acoustic\:frequency}={f}_{a}\cdot C\cdot {v}_{f}^{-1}$$

### Elastic dispersion curves

The elastic dispersion curves of the metamaterials were modelled using similar wave propagation and periodic boundary conditions to that used for modelling the acoustic dispersion curves^10^. The general undamped equation of motion under no force excitation is obtained using

5 $$Kq+M\ddot{q}=0,$$

where $K$ and $M$ are the harmonic stiffness and mass matrices, respectively and $\ddot{q}$ is the derivative of the displacement $q$. Equation (5) substitutes Eq. (1) as the governing equation for modelling the elastic dispersion curves. Using similar Floquet boundary conditions, the pressure $p$ in Eq. (2) is swapped for the displacement $q$ to approximate periodic boundary conditions. The frequencies of the elastic waves propagating within the metamaterial are obtained by solving the eigenvalue problem

6 $${{\varvec{K}}}_{{\varvec{e}}}-{{\varvec{\omega}}}^{2}{{\varvec{M}}}_{{\varvec{e}}}=0,$$

where ${{\varvec{K}}}_{{\varvec{e}}}$ is the elastic stiffness matrix and ${{\varvec{M}}}_{{\varvec{e}}}$ is the elastic mass matrix. The elastic dispersion curves are then plotted alongside the acoustic dispersion curves. The normalised elastic frequency is plotted on the $x-$axis using.

7 $$\mathrm{Normalised\:elastic\:frequency}={f}_{e}\cdot C\cdot {v}_{s}^{-1},$$

where ${f}_{e}$ is the absolute elastic frequency and ${v}_{s}$ is the speed of wave propagation in the elastic medium.

### Finite simulation of acoustic and elastic waves

A metamaterial with a finite length was modelled using free-free boundary conditions. The relative acoustic pressure response was calculated using.

8 $$\mathrm{Relative\:pressure\:response}=\mathrm{log}\left(\frac{{p}_{o}}{{p}_{i}}\right),$$

where the subscripts $o$ and $i$ denote the output and input, respectively. On the elastic simulation side, a harmonic force of 1 N was applied perpendicular to the solid surface of the metamaterial at the input as in Fig. 4. The elastic transmission was calculated using.

9 $$\mathrm{Elastic\:transmission}=20\mathrm{log}\left(\frac{{a}_{o}}{{a}_{i}}\right),$$

where $a$ is the acceleration at the output $o$ and input $i$.

### Manufacturing and experimental testing

A metamaterial with a finite length was manufactured on a LPBF system using Nylon-12 as a building material. The properties of Nylon-12 are presented in Table 2.

**Table 2:** Material properties of LPBF Nylon-12.

Density	1000 kg m^-3^
Young’s modulus	1.75 GPa

**Figure 8:**
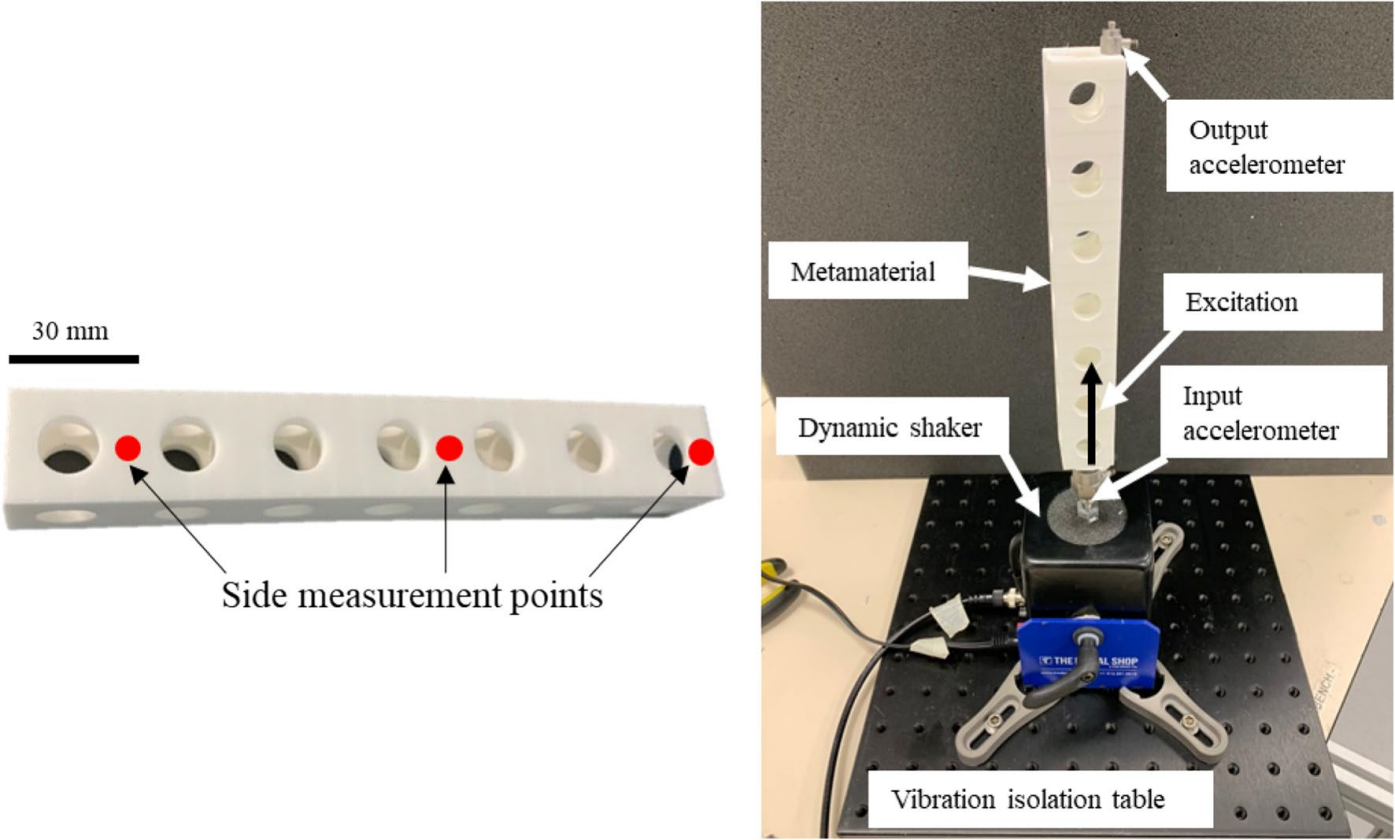
Demonstration of the experimental setup (right) and the metamaterial manufactured with LPBF (left).

The LPBF system used a laser power of 21 W with a scan speed of 2500 mm s^−1^ and hatch spacing of 0.25 mm. The nominal spot size of the laser and layer thickness was 0.3 mm and 0.1 mm, respectively. The metamaterial was manufactured successfully and is shown in Fig. 8.

To measure the experimental response, a dynamic response setup was assembled comprising a dynamic shaker (Modal Shop Shaker K2007E01), a junction box (Polytec VIB-E-400), an impedance head (PCB 288D01) and three accelerometers (PCB 352C65). Two sets of measurements were taken, longitudinal and cross-receptance. During the measurement process of the longitudinal response, the excitation signal was sent up through the bottom of the metamaterial as shown in Fig. 8. An accelerometer is placed between the dynamic shaker and the metamaterial to record the acceleration at the input. An accelerometer was attached to the top surface of the metamaterial to record the acceleration at the output. During the measurement process of the transfer-receptance response, the output accelerometer was affixed to the side of the metamaterial to record the transverse response. Three different positions were used when recording the data, after the first unit cell, third unit cell and seventh unit cell. This is to depict the evolution of the attenuation region through the metamaterial. All measurements were recorded in m s^−2^ with a normalised frequency resolution of 3.4 × 10^–5^. The magnitudes of each measurement were averaged over 24 spectral sweeps and the longitudinal and cross-receptance responses were then calculated in dB using Eq. (9).
